# Signature of immune-related metabolic genes predicts the prognosis of hepatocellular carcinoma

**DOI:** 10.3389/fimmu.2024.1481331

**Published:** 2024-11-25

**Authors:** Weibin Zhuo, Hongmei Xia, Bin Lan, Yu Chen, Xuefeng Wang, Jingfeng Liu

**Affiliations:** ^1^ Innovation Center for Cancer Research, Laboratory of Radiation Oncology and Radiobiology, Clinical Oncology School of Fujian Medical University, Fujian Cancer Hospital, Fuzhou, Fujian, China; ^2^ Huzhou Central Hospital, Fifth School of Clinical Medicine, Zhejiang Chinese Medical University, Huzhou, Zhejiang, China; ^3^ Interdisciplinary Institute for Medical Engineering, Fuzhou University, Fuzhou, Fujian, China; ^4^ XMU-Fujian Cancer Hospital Research Center of Metabolism and Cancer, Xiamen University, Xiamen, Fujian, China

**Keywords:** liver cancer, immunity, metabolism, risk score, prognostic model

## Abstract

**Introduction:**

The majority of liver cancer cases (90%) are attributed to hepatocellular carcinoma (HCC), which exhibits significant heterogeneity and an unfavorable prognosis. Modulating the immune response and metabolic processes play a crucial role in both the prevention and treatment of HCC. However, there is still a lack of comprehensive understanding regarding the immune-related metabolic genes that can accurately reflect the prognosis of HCC.

**Methods:**

In order to address this issue, we developed a prognostic prediction model based on immune and metabolic genes. To evaluate the accuracy of our model, we performed survival analyses including Kaplan-Meier (K-M) curve and time-dependent receiver operating characteristic (ROC) curve. Furthermore, we compared the predictive performance of our risk model with existing models. Finally, we validated the accuracy of our risk model using mouse models with *in situ* transplanted liver cancer.

**Results:**

By conducting lasso regression analysis, we identified four independent prognostic genes: fatty acid binding protein 6 (FABP6), phosphoribosyl pyrophosphate amidotransferase (PPAT), spermine synthase (SMS), and dihydrodiol dehydrogenase (DHDH). Based on these findings, we constructed a prognostic model. Survival analysis revealed that the high-risk group had significantly lower overall survival (OS) rates. Besides that, the ROC curve demonstrated the effective prognostic capability of our risk model for hepatocellular carcinoma (HCC) patients. Furthermore, through animal experiments, we validated the accuracy of our model by showing a correlation between high-risk scores and poor prognosis in tumor development.

**Discussion:**

In conclusion, our prognostic model surpasses those solely based on immune genes or metabolic genes in terms of accuracy. We observed variations in prognosis among different risk groups, accompanied by distinct immune and metabolic characteristics. Therefore, our model provides an original evaluation index for personalized clinical treatment strategies targeting HCC patients.

## Introduction

HCC is the most prevalent primary malignant liver tumor, exhibiting comparable rates of morbidity and mortality, which are indicative of a poor prognosis (1). Diverse therapeutic regimens are available for different stages of HCC development, encompassing liver resection, liver transplantation, transarterial chemoembolization, systemic supportive therapy, among others (2–4).

Immunotherapy is regarded as the fourth cornerstone of cancer treatment, following surgery, chemotherapy, and radiotherapy (5). Immunotherapy encompasses immune cell therapy and immune checkpoint inhibitors as its primary modalities. Immune cell therapy offers significant advantages in HCC treatment, including adoptive cell transfer therapy (ACT), chimeric antigen receptor T-cell (CAR-T) therapy and tumor-infiltrating lymphocytes (TILs) (6, 7). Additionally, immune checkpoint inhibitors have revolutionized HCC therapy with the approval of nivolumab and pembrolizumab targeting the PD-1/PD-L1 pathway for advanced HCC treatment, instilling new hope (8, 9). Notably, the combination therapy of immune checkpoint inhibitors atezolizumab and bevacizumab has demonstrated remarkable therapeutic efficacy in advanced hepatocellular carcinoma (HCC) patients, representing a pivotal breakthrough in HCC treatment (10). Moreover, considering the triumph of immunotherapy, immune-related genes may emerge as crucial prognostic indicators for both HCC development and therapeutic interventions.

In the 1920s, Otto Warburg initially reported the Warburg effect, which refers to the phenomenon wherein tumor cells exhibit a preference for glycolysis as their primary energy source, even in the presence of sufficient oxygen (11). The advantage of this metabolic adaptation lies primarily in its provision of a favorable tumor microenvironment conducive to cancer cell proliferation and rapid energy supply for accelerated tumor growth, thereby gaining a growth advantage (12). Besides aberrant glucose metabolism, abnormal lipid and amino acid metabolism also contribute significantly to cancer development (13). Numerous investigations have demonstrated that tumor metabolism is highly adaptable and reprogramming of cellular metabolism effectively influences both initiation and progression of tumors (14).

Although numerous studies have focused on predicting the prognosis of liver cancer by prioritizing immune or metabolic genes, it is insufficient to solely analyze these gene sets independently. In this study, we identified four immune-related metabolic genes whose expression levels were significantly associated with the prognosis of hepatocellular carcinoma (HCC). Subsequently, we developed a risk model incorporating these genes to accurately forecast the prognosis of HCC.

## Materials and methods

### Data acquisition and processing

Transcriptional data were obtained from TCGA database (https://www.cancer.gov/ccg/research/genome-sequencing/tcga), including 50 normal liver samples and 374 HCC samples. Microarray data profiles of GSE112790 were obtained from GEO database (https://www.ncbi.nlm.nih.gov/geo/), which included 15 normal liver samples and 183 HCC samples. Additionally, clinical data pertaining to HCC samples were acquired via TCGA and GEO databases (**Supplementary Tables 1A, B**).

### Clustering analysis

Non-negative Matrix Factorization (NMF) clustering algorithm was used to perform clustering analysis on the TCGA samples. The “brunet” option was chosen and a total of 10 iterations were performed. The number of clusters k was set from 2 to 10. The optimal clustering number was determined to use cophenetic, dispersion, and silhouette indicators, and selected as 2.

### Prognostic model construction

The differentially expressed genes (DEGs) and clinical survival were integrated from the data of TCGA and GEO databases (**Supplementary Tables 1C, D**). By using the integrated data, a random grouping was conducted. The train group consisted of 70% of the samples and the remaining 30% were assigned to the test group. To identify significant genes associated with prognosis, univariate COX correlation analysis was conducted in the train group (**Supplementary Table 2A**). To prevent excessively fitting, the least absolute shrinkage and selection operator (LASSO) analysis (ten-fold cross-validation) was employed to identify the significant predictive genes (**Supplementary Table 2B**). Subsequently, multivariate COX correlation analysis was conducted to identify prognostic gene (**Supplementary Tables 2C, D**), and then we constructed a risk model. The risk score was calculated as FABP6 × (0.220543173197653) + PPAT × (0.447449310621628) + SMS × (0.478136055547003) + DHDH × (0.230507719504424). Samples were classified into high-risk and low-risk groups using the median risk score of train group from the TCGA database.

### Validation of model accuracy

To validate if our model can accurately distinguish survival differences between different risk groups, we adopted survival analyses, including K-M curve and ROC curve, survival status heatmap and analysis of survival differences in different clinical subgroups. In addition, to investigate whether the constructed model was superior to other reported models, we compared multiple models using K-M curve, ROC curve, and C-index value.

### Evaluation of model predictive ability

By using R packages “survival”, “regplot”, and “rms”, a prognostic nomogram was constructed. The predictive ability of nomogram was assessed using decision curve analysis (DCA) and the ROC curve.

### Correlation of risk scores with clinicopathological characteristics

The clinicopathological characteristics of samples were integrated with their corresponding risk scores. The clinicopathological characteristics associated with prognosis were screened using COX correlation analysis.

### Estimation of immunotherapy response

The gene expression data and immune cell infiltration information derived from TCGA database were merged with their corresponding the risk scores (**Supplementary Table 3A**), and then we analyzed the correlation between the risk score and the expression levels of immune checkpoint-related genes (ICRGs), as well as between risk score and immune cell infiltration. Correlation plot was generated using R packages “ggpubr” and “corrplot”.

### Enrichment analysis

The gene expression data of different risk groups was subjected to the Gene Set Enrichment Analysis (GSEA) with R package “clusterProfiler” (**Supplementary Table 3B**), and then we selected top five pathways that showed significant enrichment for each group. The “c2.cp.kegg. Hs.symbols” gene set was obtained from the Molecular Signatures Database (https://www.gsea-msigdb.org/gsea/msigdb) for further analysis.

### Cell culture and RNA extraction

Hepa1-6 cells were cultured in DMEM (Gibco), and H22 cells were cultured in RPMI 1640 (Gibco), both of which were supplemented with 10% FBS (Biochannel) and 1% streptomycin - penicillin (Gibco), and then placed in the 37°C incubator containing 5% CO_2_. When cell density was 70% - 80%, the culture medium was discarded and the cells were washed twice with phosphate buffer solution (PBS). The TRIzol reagent (Invitrogen) was used to lyse cells for 5 minutes and then 0.2 mL of trichloromethane (Chinese medicine Hushi) was added to every 1mL of TRIzol reagent, then the mixture was shaken vigorously for 15 seconds and left to stand at room temperature for 5 minutes. Centrifuged cell lysate at 4°C and 12700 rpm for 15 minutes, and then mixed upper aqueous phase with equal volume isopropanol (Chinese medicine Hushi). Gently inverted and mixed well, then rested for 5 minutes. The mixture was then centrifuged mixture at 4°C and 12700 rpm for 3 minutes. After discarding the supernatant and washing with 75% ethanol (Chinese medicine Shanghai trial), we centrifuged again and discarded the supernatant. After opening the lid and allowing to stand for 2 minutes, RNA precipitation was dissolved by 30 -100 µL DEPC water.

### Fluorescence quantitative PCR

To obtain cDNA, the ReverTra Ace qPCR RT Kit (TOYOBO) was utilized for RNA reverse transcription. Then 2X Universal SYBR Green Fast qPCR Mix (ABclonal) was applied for qPCR. The gene expression level was quantified using the 2^-ΔCt^ method and each sample was tested in triplicate. The corresponding primers for the four genes could be found in the **Supplementary Materials** (**Supplementary Table 4**).

### Orthotopic mouse model analysis

To investigate the sensitivity of tumors with different risk scores to metformin treatment, orthotopic mouse models were established using Hepa1-6 and H22 liver cancer cells. Six-week-old male BALB/c and C57BL/6J mice were obtained from GemPharmatech LLC. The mice were maintained under pathogen-free conditions and were provided with sterilized food and water. C57BL/6J mice were inoculated with Hepa1-6 cells and BALB/c mice with H22 cells. There were 12 mice of each strain. First, 1x10^6^ cells were injected into the liver of each mouse with an injection volume of 40 µL. Thereafter, each strain was randomly divided into an experimental group and a control group. On the third day, oral gavage treatment started, and the experimental group was given metformin (250mg/kg/day, Cat: M21704, HARVEYBIO) dissolved in physiological saline with a dosage of 100 µL. The control group was given an equal amount of physiological saline. After 2 weeks, the mice were euthanized and the livers were harvested.

### Hematoxylin-eosin staining

Firstly, the liver slices were dried in a 65°C oven for 30 minutes and then followed by immersing in xylene I, II and III for 5 minutes each. After immersing the slices in anhydrous ethanol I and II for 1 minute each, they were soaked in 95% ethanol I and II for 1 minute each. The slices were rinsed with water for 1 minute, then stained with hematoxylin solution (Baso) for 5 minutes, and then rinsed with water for 10 minutes. Next, the slices were stained with eosin aqueous solution (ZSGB-BIO) for 1 minute, then soaked in 95% ethanol III and IV for 1 minute each, followed by soaking in anhydrous ethanol III and IV for 1 minute each. Finally, the slices were soaked in xylene IV and V for 1 minute each and sealed with neutral resin.

### Statistical analysis

The study was analyzed using R version 4.2.1. Immune cell infiltration level and immune checkpoint gene expression were analyzed by Wilcoxon test. The comparison of risk scores between the Hepa1-6 and H22 liver cancer cells and the difference analysis of the ratio of liver weight to body weight were statistically analyzed using an unpaired t-test. Unless specifically annotated otherwise, a two-tailed *P*-value of less than 0.05 was considered to indicate statistical significance in this study.

## Results

### Differentially expressed genes shape prognosis and tumor immunity

**Figure 1:**
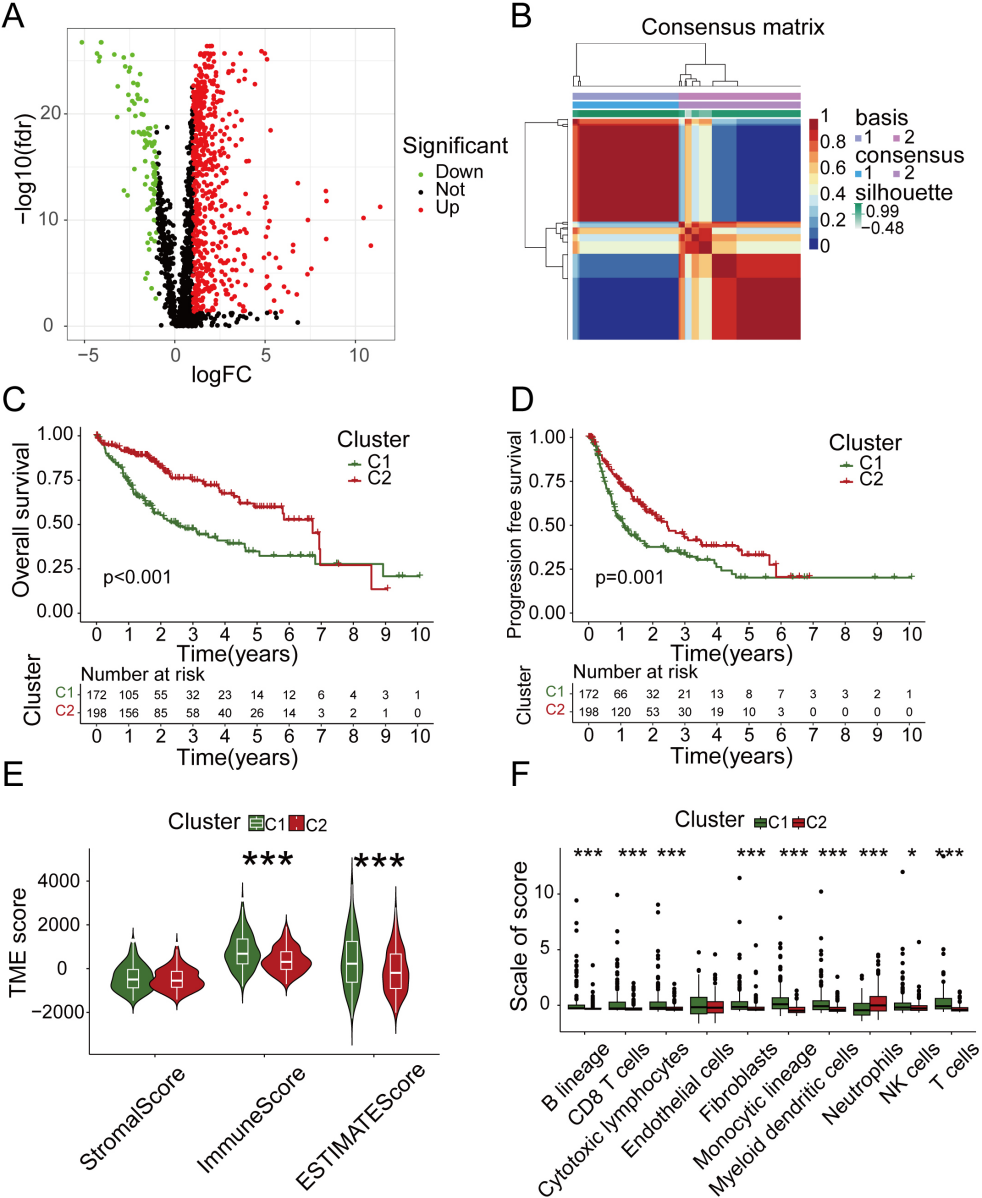
NMF subtyping and analysis of tumor microenvironment. **(A)** Volcano map of differentially expressed genes in normal and tumor samples. **(B)** Two subgroups were identified as optimal values for consensus clustering. **(C)** OS analysis of two subtypes. **(D)** PFS analysis of two subtypes. **(E)** Tumor microenvironment related scores of two subtypes. **(F)** The immune infiltration analysis of two subtypes.

To understand the overall effect of immune genes and metabolic genes in HCC prognosis, we conducted DEGs analysis on normal and tumor samples (**Figure 1A**) and performed NMF subtyping based on the analysis results (**Supplementary Figure 1A**). Based on the cophenetic correlation analysis (**Supplementary Figure 1B**), the optimal k value was determined to be 2, so we divided all samples into two types, C1 and C2 (**Figure 1B**). Subsequently, we analyzed the DEGs between different subtypes (**Supplementary Figure 1C**). Our analyses showed that the OS and progression-free survival (PFS) of C2 exhibited higher levels compared to C1 (**Figures 1C, D**).To discern the differences in the tumor immune microenvironment (TIME) among samples with different subtypes, we conducted an analysis of TIME. Interestingly, the analysis revealed that C1 had higher immune cell score and comprehensive score compared to C2 (**Figure 1E**). To further investigate which immune cells had different expression levels in different subtypes, we subsequently performed an immune cell infiltration analysis. We found that the neutrophil distribution in C2 group was higher than C1, but this was not the case for CD8^+^ T cells, cytotoxic lymphocytes, NK cells or other T cells (**Figure 1F**). Therefore, both the quantity and quality of anti-tumor immune cells determined their effects on tumor prognosis.

### High expression of prognosis-related genes elevates mortality

**Figure 2:**
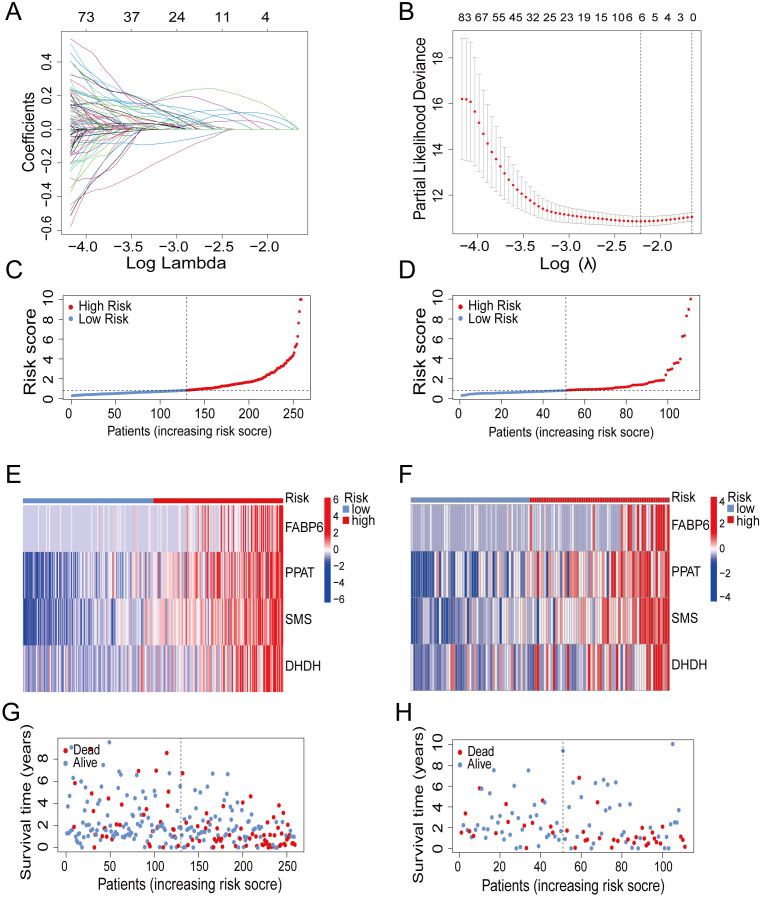
Construction of prognostic model. **(A)** Partial likelihood deviance with changing of log (λ) plotted through LASSO Cox regression in 10-fold cross-validations. **(B)** Coefficients with changing of log (λ) plotted through LASSO Cox regression in 10-fold cross-validations. **(C, D)** The distribution of risk scores in the train group and test group. **(E, F)** Heatmap of 4 genes expression in the train group and test group. **(G, H)** The survival status of patients in the train group and test group.

To obtain prognosis-related genes (PRGs), we conducted univariate COX correlation analysis within the train group, followed by lasso regression analysis and cross validation (**Figures 2A, B**).Then, we screened out four independent PRGs, including fatty acid binding protein 6 (FABP6), phosphoribosyl pyrophosphate amidotransferase (PPAT), spermine synthase (SMS) and dihydrodiol dehydrogenase (DHDH). The aforementioned factors all play a pivotal role in metabolic and immune regulation and exhibit significant correlations with survival outcomes.

FABP6 is overexpressed in various cancers. Inhibiting its expression can halt the cell cycle and boost the secretion of immune response-related chemokines, facilitating CD8^+^ T cell recruitment (15, 16). The enzyme PPAT facilitates the conversion of glutamine into phosphoribosylamine, which is recognized as an immunomodulatory nutrient and exhibits a rapid increase in uptake upon activation of naive T cells (17). The increased SMS expression in HCC is linked to a negative prognosis and can hinder the effectiveness of immune checkpoint blockade therapy (18). DHDH catalyzes the conversion of NADP^+^ to NADPH, which is a substrate for generating reactive oxygen species (ROS). These ROS can affect dendritic cell maturation and cross-presentation capabilities, as well as T cell immune response, thereby modulating immune reactions (19, 20). Elevated DHDH expression in cancer has been correlated with unfavorable prognostic outcome (21–23).

To fully encompass the potential prognostic significance of immune and metabolic genes in HCC, we visualized the gene coefficient. Finally, we established a prognostic model by utilizing the expression levels and corresponding coefficient of the PRGs. The different risk groups were divided according to median risk score of train group (**Figure 2C**). We adopted the same approach in the test group (**Figure 2D**). The expression of four genes in high-risk group were much more than the low-risk group (**Figures 2E, F**). Correspondingly, patients in high-risk group showed an increased mortality (**Figures 2G, H**).

### Risk model effectively predicts the survival rates of HCC patients

**Figure 3:**
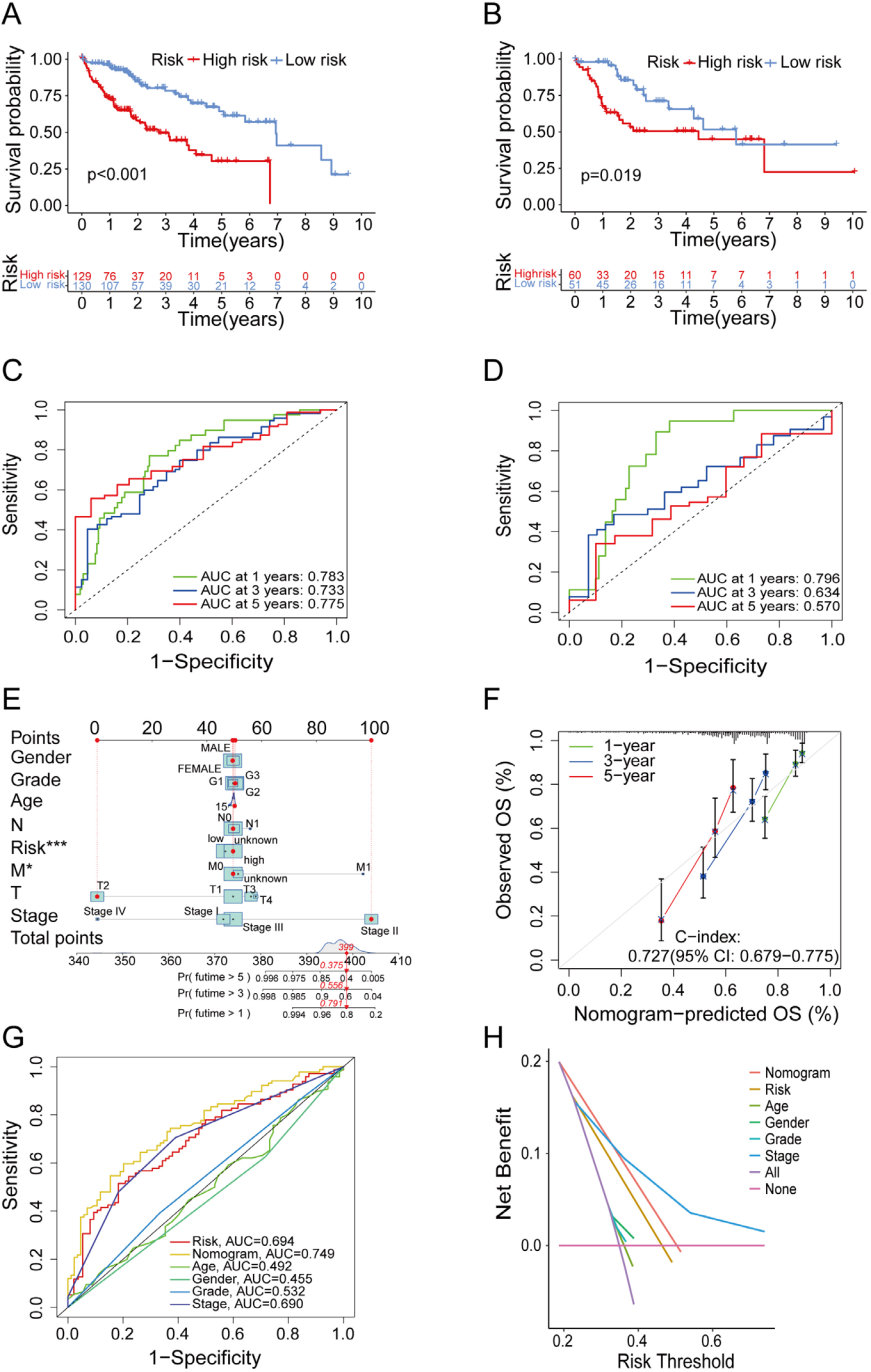
Verification of the predictive ability of the model. **(A, B)** Kaplan–Meier curves of survival in train group **(A)** and test group **(B)**. **(C, D)** Time-dependent ROC curve of the risk score model for predicting 1, 3 and 5 years in train group **(C)** and test group **(D)**. **(E)** The nomogram for predicting survival proportion of patients in 1, 3 and 5 years. **(F)** The calibration plots for predicting patient survival at 1, 3 and 5 years. **(G)** Comparison of time-dependent ROC curve of multiple factors. **(H)** Comparison of decision curve analysis of multiple factors.

Through the survival analysis of different risk groups in train groups and test groups, our study found that high-risk group showed significantly reduced OS compared to low-risk group (**Figures 3A, B**). Besides that, a survival analysis based on all TCGA and GEO samples also showed the same results (**Supplementary Figures 2A, B**). Accordingly, our model could effectively distinguish high- and low-risk group. Additionally, the ROC curve showed that the area under curve (AUC) of the train group was approximately 0.783, 0.733 and 0.775 for 1, 3 and 5 years (**Figure 3C**). The AUC of the test group was approximately 0.796, 0.634 and 0.570 for 1, 3 and 5 years (**Figure 3D**). In summary, the above findings indicated that risk model had efficient predictive power for HCC survival.

In order to evaluate predictive accuracy of the risk model and clinical characteristics, and to confirm whether the risk model could serve as an independent prognostic factor, we conducted an analysis of the predictive association among age, gender, tumor stages, grades, and the risk score, considering individual factor as well as multiple factors. The findings denoted that the risk score served as an independent predictive factor of HCC survival (**Supplementary Figures 2C, D**). Subsequently, we combined risk score with age, gender and pathological stage to establish a prognostic nomogram (**Figure 3E**). As shown by the calibration curve (**Figure 3F**), the nomogram could predict OS with relatively great accuracy. The ROC curves of several indicators (**Figure 3G**) and DCA (**Figure 3H**) revealed that the nomogram exhibited superior predictive ability compared to the indicator of age, gender, grade and stage. In summary, the above results validated the predictive potential of the nomograms in predicting HCC prognosis.

### Risk model exhibits potent clinical applicability

**Figure 4:**
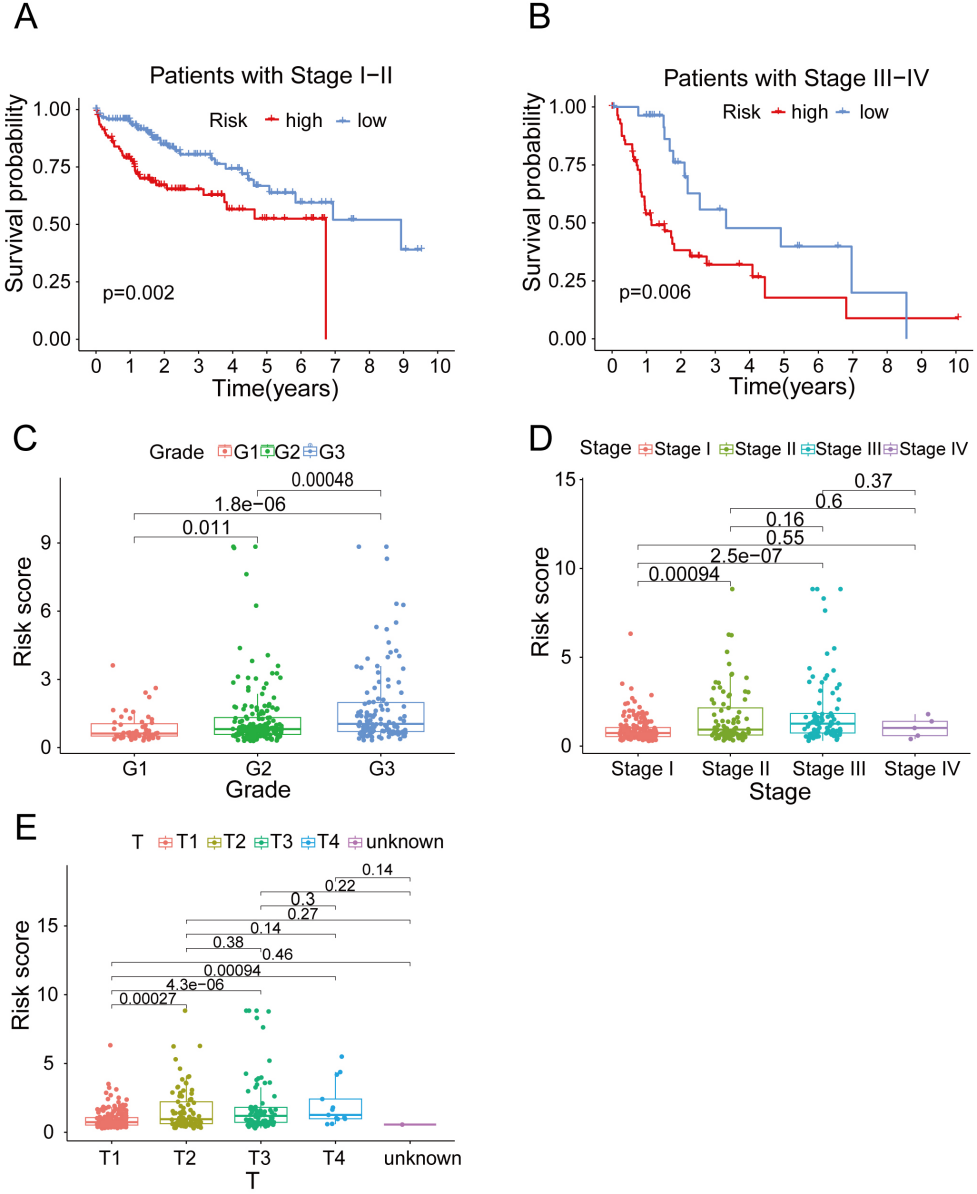
The clinical correlation analysis. **(A)** OS in the high-risk and low- risk groups of HCC patients in stage I-II. **(B)** OS in the high-risk and low- risk groups of HCC patients in stage III-IV. **(C)** The clinical correlation analysis of grade. **(D)** The clinical correlation analysis of cancer stage. **(E)** The clinical correlation analysis of T stage.

In order to ascertain the suitability of the model for patients in various clinical groups, we analyzed the survival of patients in different clinical stages. Our findings suggested that, regardless of whether the patients had early or advanced HCC, the model exhibited high accuracy in distinguishing high-risk and low-risk patients (**Figures 4A, B**). To investigate variations in risk scores among patients in distinct clinical categories, we conducted a clinical correlation analysis. Our analysis revealed a positive correlation between the risk score and tumor characteristics, including tumor grade, stage and T stage (**Figures 4C–E**). As expected, the predictive function of the risk score was not affected by age, gender, M stage or N stage (**Supplementary Figures 3A–D**).

### The immune status and GSEA enrichment pathways differ between the two risk groups

Immunotherapy for HCC has emerged as a prominent research focus in recent years. In an effort to explore the correlation between immune checkpoints, immune cells and patient prognosis, we conducted correlation analysis. In the high-risk group, a significant proportion of immune checkpoints (92%, 11 out of 12) and immune cell types (70%, 7 out of 10) exhibited high expression levels (**Supplementary Figures 4A, B**). The aforementioned findings suggested that patients classified in high-risk group may potentially derive greater therapeutic benefits from immunotherapy. In order to identify active molecular functions and pathways in different risk groups, we performed GSEA. Molecular biological processes, such as cell cycle, ECM receptor interactions, hematopoietic cell lines and neuroactive ligand-receptor interaction, were primarily enriched in the high-risk groups (**Supplementary Figure 4C**). Metabolic pathways, such as β-alanine metabolism, fatty acid metabolism, tryptophan metabolism and primary bile acid biosynthesis, were found to be enriched in the low-risk group (**Supplementary Figure 4D**).

### The predictive power of the risk model is higher compared to that of other tested models

**Figure 5:**
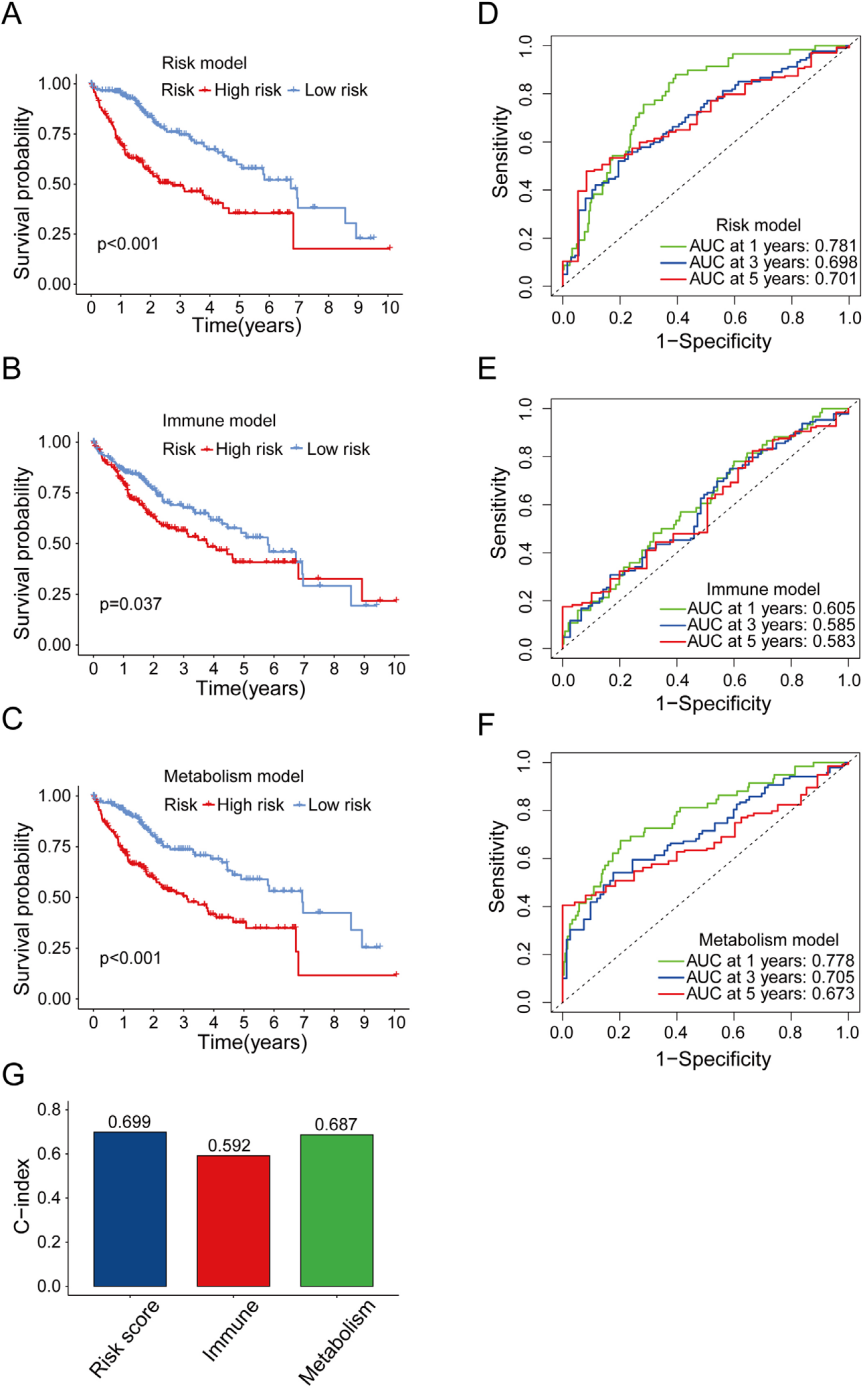
Comparison between risk model and other prognostic models. **(A)** OS analysis of high-risk and low-risk groups in risk model. **(B)** OS analysis of high-risk and low-risk groups in immune model. **(C)** OS analysis of high-risk and low-risk groups in metabolism model. **(D-F)** Time-dependent ROC curve of the risk model **(D)**, immune model **(E)** and metabolism model **(F)** for predicting 1, 3 and 5 years. **(G)** Concordance index comparison of three prognostic models.

To determine the predictive power of our risk model compared to that of previously reported models, we conducted OS analyses (**Figures 5A–C**) and ROC curve (**Figures 5D–F**). We found that the AUC of our model was overall better than that of the other models. In addition, similar results were observed when we used the C-index method to analyze each model (**Figure 5G**). In conclusion, the aforementioned finding suggested that the model we had constructed was superior to other prognostic models, which were based solely on immune genes or metabolic genes, in terms of accuracy.

### Higher risk scores lead to decreased sensitivity to metformin in liver cancer animal models

**Figure 6:**
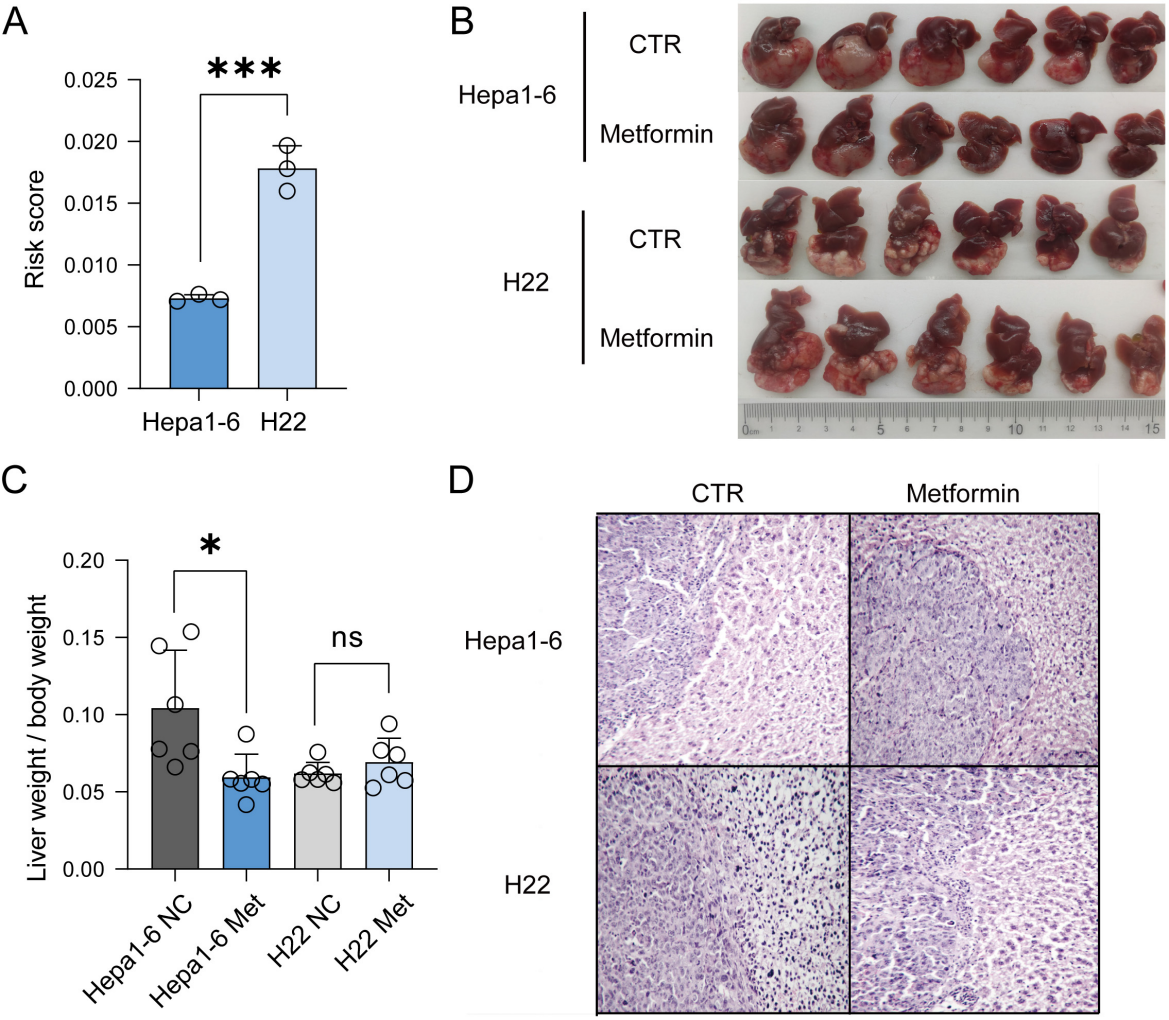
Verification of the accuracy of the model *in vivo* experiments. **(A)** Risk score of Hepa1-6 and H22 liver cancer cells. **(B)** Using Hepa1-6 and H22 cells to establish orthotopic mouse models. **(C)** Comparison of liver weight to body weight between the two models. **(D)** H&E staining indicated that after treatment with metformin, the prognosis of H22 liver cancer was worse than that of Hepa1-6 liver cancer.

To further verify the relationship between the risk score and prognosis under metabolism targeting treatment, we performed qPCR analysis on the expression levels of four genes in Hepa1-6 and H22 liver cancer cells (**Supplementary Figures 5A, B**; **Supplementary Table 5**), and calculated their risk scores, with Hepa1-6 cells scoring about 0.00730 and H22 cells scoring about 0.0178 (**Figure 6A**). This indicated that the risk value of H22 liver cancer cells was approximately twice that of Hepa1-6 liver cancer cells. Therefore, we supposed that the prognosis of H22 liver cancer would be worse than that of Hepa1-6 liver cancer. Research has revealed that metformin has therapeutic effects on various cancers, including liver cancer. Therefore, we further investigated the therapeutic effects of metformin on mice with Hepa1-6 and H22 orthotopic tumors, and observed the prognosis. The results depicted that, compared with the Hepa1-6 model, metformin had a poor therapeutic effect on the H22 model (**Figures 6B, C**; **Supplementary Table 6**). The H&E staining revealed that the arrangements of tumor cells in both models were irregular, with diverse cell morphologies and increased nuclear division. After treatment with metformin, the morphology of tumor cells in the Hepa1-6 model improved, while there was no significant change in the H22 model (**Figure 6D**). In summary, the higher the risk score, the lower the response to metformin treatment in liver cancer.

## Discussion

HCC ranks as the second most common cause of cancer-related deaths globally (24), characterized by an estimated five-year survival rate of around 18% and a poor prognosis (25). Metabolism pattern determined the immunocyte fate and their regulation on tumor (26–28). Over the past few years, significant progress has been made in management of HCC, including in-depth research on tumor metabolism reprogramming (29–32) and important breakthroughs in immunotherapy (33–35). Given the significant role of metabolism and immunity in the incidence, progression, and management of HCC, it’s necessary and feasible to predict HCC prognosis using genes involved in immune and metabolic processes. In this study, we identified four gene markers, FABP6, PPAT, SMS, and DHDH, which are composed of immune-relevant metabolic genes. A risk model was designed utilizing these genes, enabling accurate prediction of HCC prognosis.

Our research has demonstrated that these genes serve as independent prognostic markers for hepatocellular carcinoma (HCC). Inhibition of FABP6 impedes the cell cycle and enhances the secretion of chemokines involved in immune response, thereby facilitating the recruitment of CD8^+^ T cells (15, 16). PPAT catalyzes the conversion of glutamine into phosphoribosylamine, a well-recognized immunomodulatory nutrient whose uptake rapidly increases upon activation of naive T cells (17). Elevated expression of SMS in HCC is associated with an unfavorable prognosis and can attenuate the efficacy of immune checkpoint blockade therapy (18). DHDH catalyzes the conversion of NADP^+^ to NADPH, which serves as a substrate for generating reactive oxygen species (ROS) that subsequently influence dendritic cell maturation and cross-presentation capabilities, as well as T cell immune responses, thus modulating overall immune reactions (19, 20). Increased DHDH expression in cancer has been correlated with an adverse prognostic outcome (21–23).

We utilized gene expression data from the TCGA and GEO databases and employed univariate COX correlation analysis and lasso regression approaches. Then, we identified four independent genes with a significant association with HCC prognosis. Subsequently, we constructed a risk model by using these genes. The survival analyses indicated that the risk model had potential to effectively differentiate high-risk and low-risk patients. To enhance the precision of model prediction, our study combined the prognostic risk score with clinical features to construct an OS prediction nomogram. Through several analyses, such as ROC curve and DCA, we found that the nomogram showed better predictive ability for the prognosis of HCC compared to other indicators. Additionally, we observed a notable positive correlation between the risk score and the tumor characteristics, including grade, stage and T stage. This finding enhances the predictive accuracy and clinical relevance of risk model.

Immunotherapy is progressively gaining significance in HCC treatment. The research on immune cells and immune checkpoints has been constantly innovating and improving. Our study found expression of immunocyte infiltration and immune checkpoint-associated genes in the high-risk group were elevated, indicating that immunotherapy may achieve ideal results in the high-risk group. In recent years, studies on prognostic model for predicting HCC outcomes have become increasingly extensive and refined. By comparing with other models (36, 37), we verified the advantages of the combined prognostic model of immune and metabolism, providing new ideas and methods for future research.

The model we have constructed has high accuracy and strong predictive potential for prognosis of HCC patients, which is the innovation and significance of this study. Nevertheless, there are also certain limitations. Firstly, the gene expression data we used to construct the model comes from a public database, so the sample size and patient treatment history cannot be fully guaranteed. Secondly, this study focuses on the impact of immune-related metabolic genes on prognosis in HCC, so the applicability of the model is somewhat limited. In the future, more clinical samples and gene expression data will be needed to support the potential clinical application of this prognostic analysis model.

## Data Availability

The raw data supporting the conclusions of this article will be made available by the authors, without undue reservation.

## Supplementary material

Supplementary Figure 1 Clustering of molecular subgroup and analysis of DEGs. **(A)** The results of NMF subtyping. **(B)** NMF rank survey. **(C)** Heatmap of the DEGs in C1 and C2 subtypes.

Supplementary Figure 2 The risk score independently predicts the survival rate of HCC. **(A)** Survival analysis of different risk groups in TCGA samples. **(B)** Survival analysis of different risk groups in GEO samples. **(C)** The univariate Cox regression analysis of the associations between the risk scores and clinical parameters and the OS of patients. **(D)** The multivariate Cox regression analysis of the associations between the risk scores and clinical parameters and the OS of patients.

Supplementary Figure 3 Clinical applicability verification of the model. **(A)** Correlation between risk score and age. **(B)** Correlation between risk score and gender. **(C)** Correlation between risk score and M stage. **(D)** Correlation between risk score and N stage.

Supplementary Figure 4 Analysis of risk score and immune status and related pathways. **(A)** Box plots of immune checkpoint molecule expression in high- and low-risk groups. **(B)** The violin plot of immune cell infiltrating in high- and low-risk groups. **(C)** The top five pathways enriched in the high-risk group. **(D)** The top five pathways enriched in the low-risk group.

Supplementary Figure 5 Expression levels of four genes in HCC cells. **(A)** Expression levels of four genes in Hepa1-6 liver cancer cells. **(B)** Expression levels of four genes in H22 liver cancer cells.
