# Supplementary figures and images for: Signature of immune-related metabolic genes predicts the prognosis of hepatocellular carcinoma
Source: Front Immunol. 2024 Nov 25;15:1481331. doi: 10.3389/fimmu.2024.1481331 (PMC11625796; doi:10.3389/fimmu.2024.1481331)

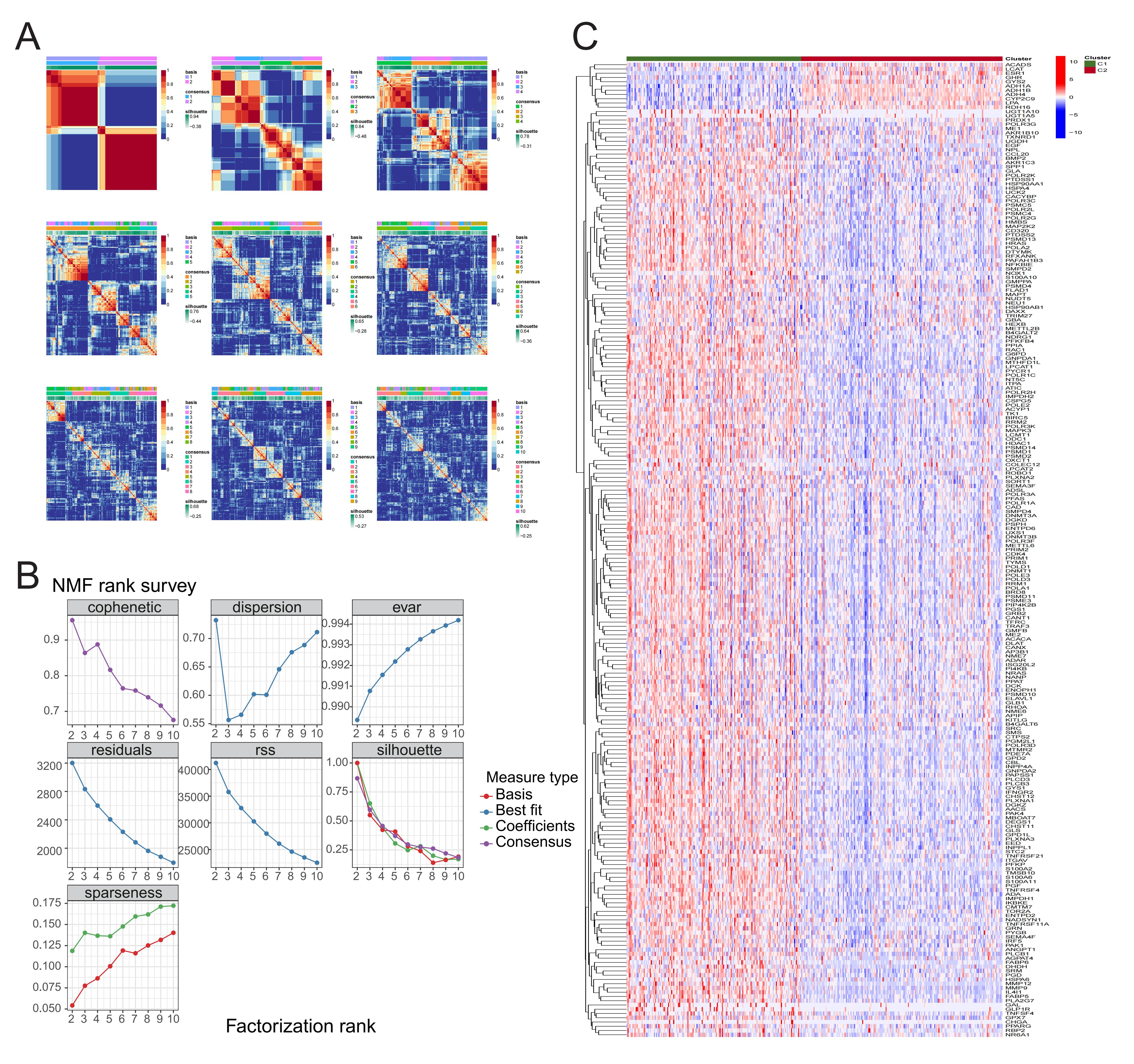

Supplement: Supplementary Figure 1 — Clustering of molecular subgroup and analysis of DEGs. (A) The results of NMF subtyping. (B) NMF rank survey. (C) Heatmap of the DEGs in C1 and C2 subtypes. [file Image1.jpg]

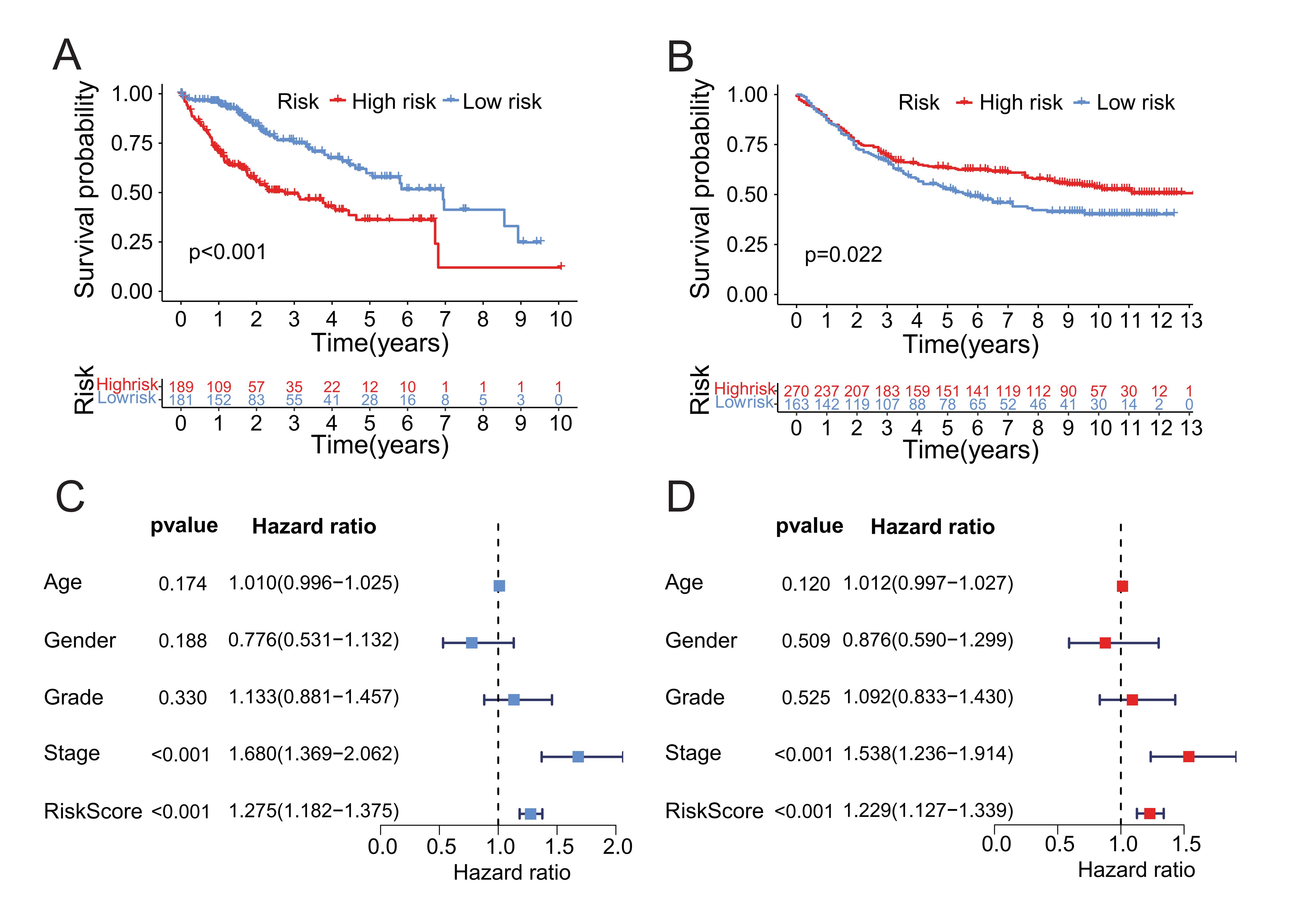

Supplement: Supplementary Figure 2 — The risk score independently predicts the survival rate of HCC. (A) Survival analysis of different risk groups in TCGA samples. (B) Survival analysis of different risk groups in GEO samples. (C) The univariate Cox regression analysis of the associations between the risk scores and clinical parameters and the OS of patients. (D) The multivariate Cox regression analysis of the associations between the risk scores and clinical parameters and the OS of patients. [file Image2.jpg]

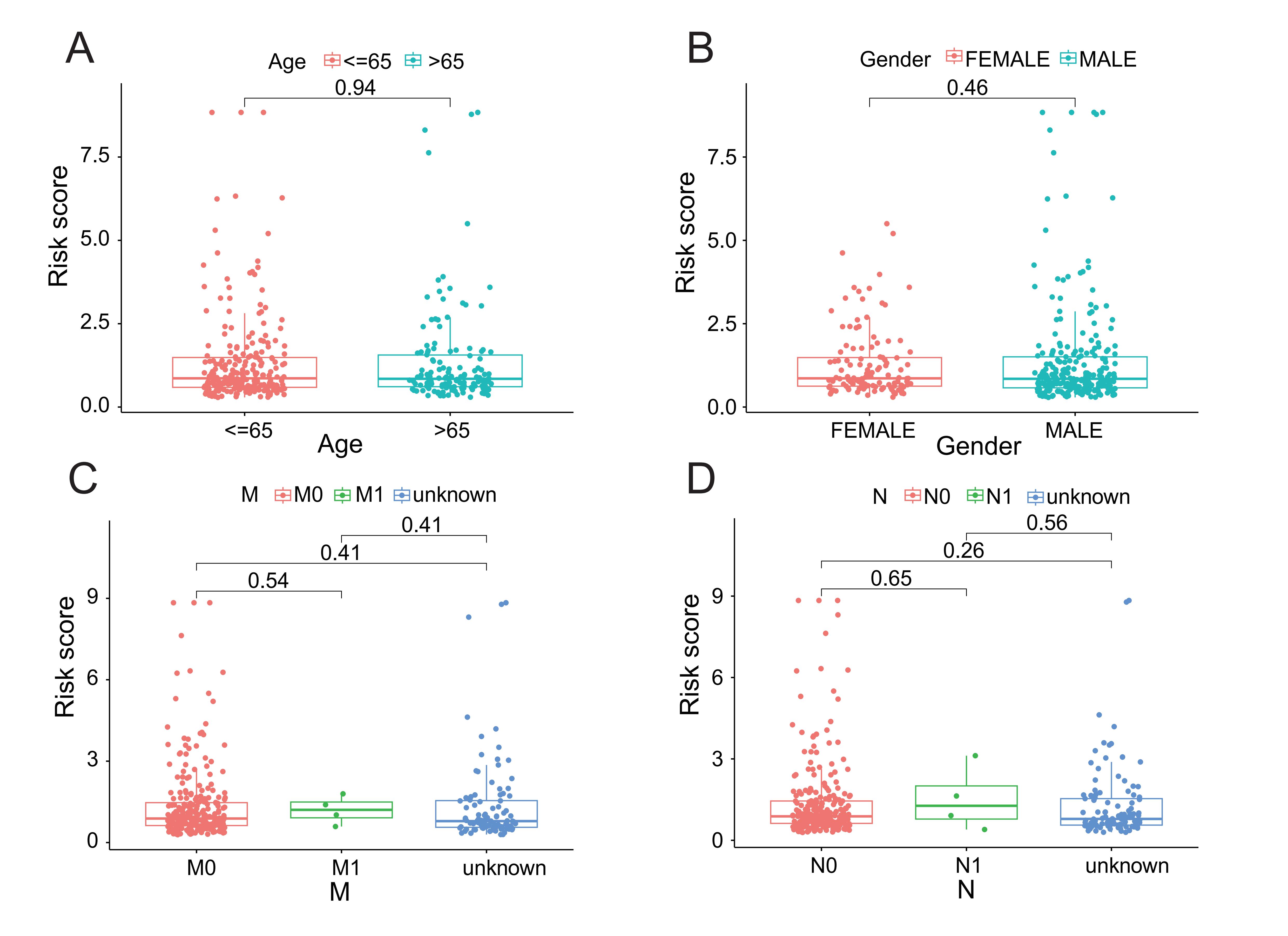

Supplement: Supplementary Figure 3 — Clinical applicability verification of the model. (A) Correlation between risk score and age. (B) Correlation between risk score and gender. (C) Correlation between risk score and M stage. (D) Correlation between risk score and N stage. [file Image3.jpg]

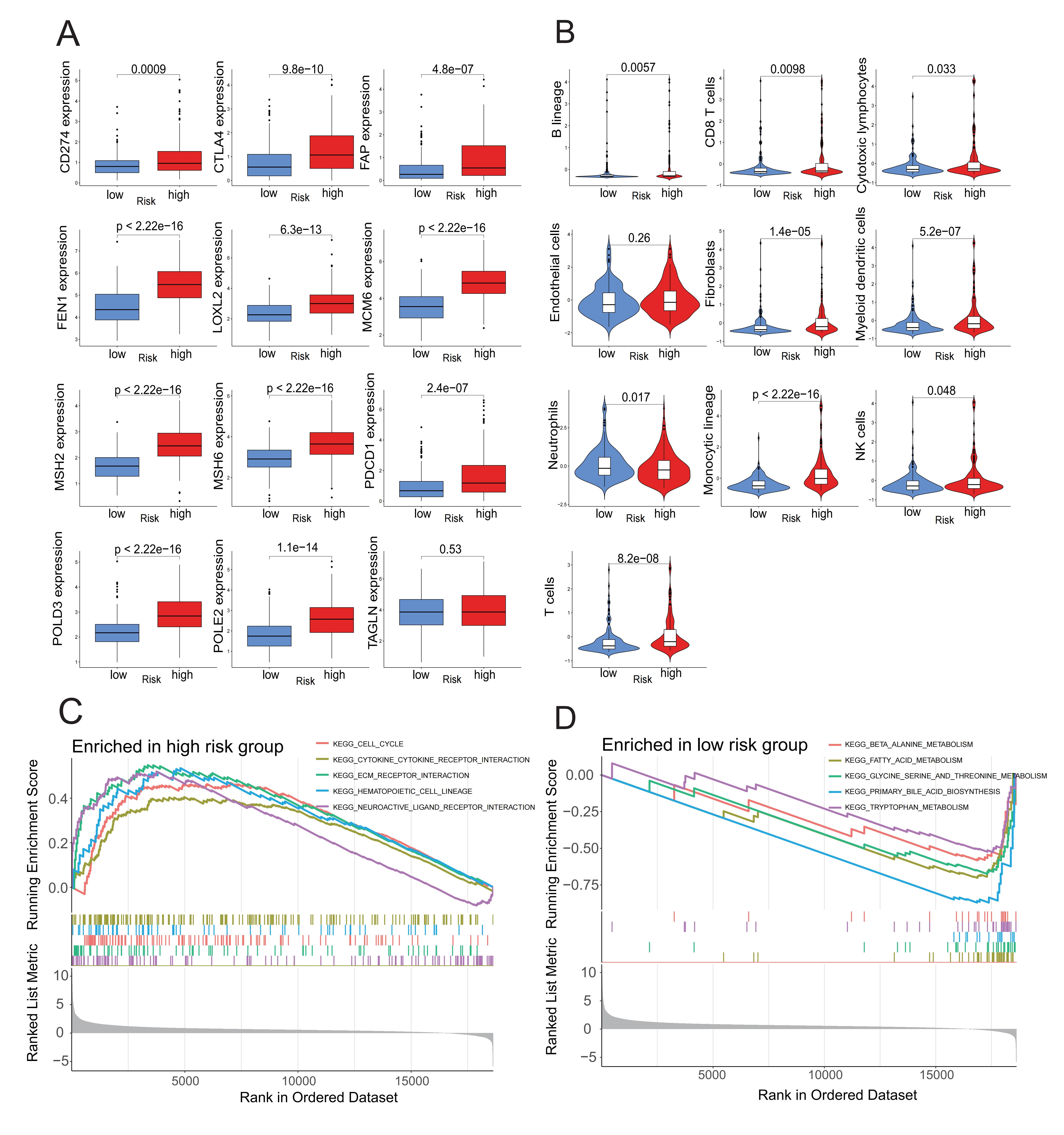

Supplement: Supplementary Figure 4 — Analysis of risk score and immune status and related pathways. (A) Box plots of immune checkpoint molecule expression in high- and low-risk groups. (B) The violin plot of immune cell infiltrating in high- and low-risk groups. (C) The top five pathways enriched in the high-risk group. (D) The top five pathways enriched in the low-risk group. [file Image4.jpg]

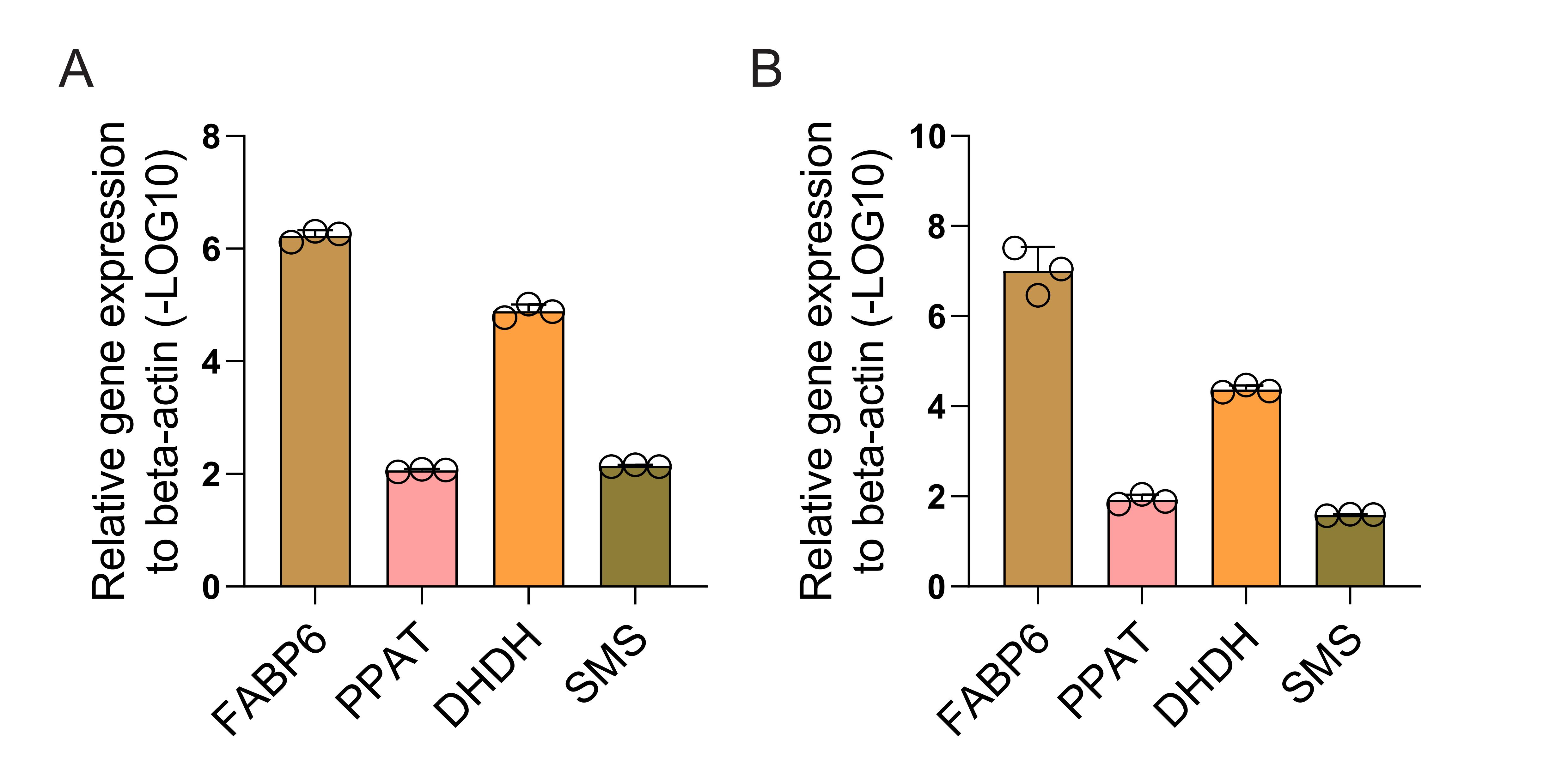

Supplement: Supplementary Figure 5 — Expression levels of four genes in HCC cells. (A) Expression levels of four genes in Hepa1-6 liver cancer cells. (B) Expression levels of four genes in H22 liver cancer cells. [file Image5.jpg]
